# Integrated Multi-Omics Analysis to Reveal the Molecular Mechanisms of Inflorescence Elongation in *Medicago sativa*

**DOI:** 10.3390/ijms25126497

**Published:** 2024-06-12

**Authors:** Xiuzheng Huang, Lei Liu, Xiaojing Qiang, Yuanfa Meng, Zhiyong Li, Fan Huang

**Affiliations:** Institute of Grassland Research of Chinese Academy of Agricultural Sciences, Hohhot 100081, China; 82101225222@caas.cn (X.H.); mengyuanfa@caas.cn (Y.M.); lizhiyong01@caas.cn (Z.L.); huangfan@caas.cn (F.H.)

**Keywords:** alfalfa, inflorescence elongation, multi-omics, functional genes

## Abstract

The morphological architecture of inflorescence influences seed production. The regulatory mechanisms underlying alfalfa (*Medicago sativa*) inflorescence elongation remain unclear. Therefore, in this study, we conducted a comparative analysis of the transcriptome, proteome, and metabolome of two extreme materials at three developmental stages to explore the mechanisms underlying inflorescence elongation in alfalfa. We observed the developmental processes of long and short inflorescences and found that the elongation capacity of alfalfa with long inflorescence was stronger than that of alfalfa with short inflorescences. Furthermore, integrative analysis of the transcriptome and proteome indicated that the phenylpropanoid biosynthesis pathway was closely correlated with the structural formation of the inflorescence. Additionally, we identified key genes and proteins associated with lignin biosynthesis based on the differential expressed genes and proteins (DEGs and DEPs) involved in phenylpropanoid biosynthesis. Moreover, targeted hormone metabolome analysis revealed that IAA, GA, and CK play an important role in the peduncle elongation of alfalfa inflorescences. Based on omics analysis, we detected key genes and proteins related to plant hormone biosynthesis and signal transduction. From the WGCNA and WPCNA results, we furthermore screened 28 candidate genes and six key proteins that were correlated with lignin biosynthesis, plant hormone biosynthesis, and signaling pathways. In addition, 19 crucial transcription factors were discovered using correlation analysis that might play a role in regulating candidate genes. This study provides insight into the molecular mechanism of inflorescence elongation in alfalfa and establishes a theoretical foundation for improving alfalfa seed production.

## 1. Introduction

The inflorescence architecture of flowering plants displays great diversity in nature and includes racemes, spikes, and capitates, which determine flower growth and seed production [1,2]. This is an effective way to investigate the potential mechanisms of inflorescence development to improve the seed yield of plants and crops [3,4,5]. Many small flowers emerge on the inflorescence peduncle, and the elongation capacity of the inflorescence peduncle affects raceme length and eventually influences flower and seed development [6]. Alfalfa (*Medicago sativa*) is an important forage, food, and Chinese herbal medicine planted worldwide [7]. The inflorescence architecture of alfalfa is a typical raceme, and its inflorescence length is a crucial factor affecting seed production [8]. The length of the inflorescence is the structural basis for the growth of more florets, and the number of florets is positively correlated with the seed yield of alfalfa [9]. The insufficient production of alfalfa seeds has limited the long-term development of the alfalfa industry. Breeders select a long-spike alfalfa cultivar based on the length of the inflorescence to increase the seed output of alfalfa, which provides an important germplasm resource for alfalfa. However, the molecular mechanisms underlying alfalfa inflorescence elongation have rarely been reported.

Lignin is an important component of plant secondary cell walls and is distributed in the cell walls of both supportive and conductive tissues [10]. Lignin hardens the cell wall by forming interwoven webs, allowing for the xylem to remain highly rigid; this allows for lignin to carry the weight of aerial structures [11]. Lignin is an essential metabolite for the development of vascular tissues; many studies have demonstrated that the accumulation and distribution of lignin affect the elongation and growth of plant organs [12,13]. Additionally, lignin precursors are produced by the phenylpropanoid biosynthesis pathway; some crucial enzymes are involved in the synthesis of lignin, including *PAL* (L-phenylalanine ammonia-lyase), *C4H* (cinnamate 4-hydroxylase), *4CL* (4-coumarate: CoA ligase) *HCT* (hydroxycinnamoyl CoA: shikimate hydroxycinnamoyl transferase), *CCoAOMT* (caffeoyl-CoA O-methyltransferase), *CCR* (cinnamoyl-CoA reductase), *CAD* (cinnamyl alcohol dehydrogenase), *F5H* (ferulate 5-hydroxylase), *COMT* (caffeic acid/5-hydroxyconiferaldehyde 3-O-methyltransferase), and *POX*/*LAC* (peroxidase/laccase) [14,15]. In alfalfa, the inflorescent peduncle is the carrier for floret development that continuously synthesizes lignin during peduncle growth. Therefore, investigating lignin biosynthesis in the peduncle may reveal the molecular mechanisms of inflorescence elongation in alfalfa.

Plant hormones play an important role in regulating lignin biosynthesis, organic matter accumulation, and other physiological activities and eventually influence the growth and development of plant tissues and organs [16,17,18]. Auxins (IAA) are frequently associated with stem elongation and root development and can promote or inhibit apical dominance [19,20]. Gibberellin (GA), one of the plant hormones necessary for plant growth, plays a role in stem development, plant flowering, seed germination, and other metabolisms [21,22]. CK can induce cell division and elongation and promote tissue differentiation, and the interaction between cytokinins and auxins plays a critical role in regulating xylem development [23,24]. Moreover, other phytohormones, such as abscisic acid (ABA), jasmonate (JA), salicylic acid (SA), and ethylene (ETH), are closely connected with participating in plant growth and development [25,26,27].

Multi-omics techniques have been widely applied to understand the mechanisms underlying inflorescence growth and development [28,29]. For example, Weng et al. investigated the transcriptional profiling between the two major inflorescence ecotypes in *Panicum hallii* based on the global transcriptome analysis; the result suggested the underlying effect of cytokinin signaling in heterochronic changes and provided some novel insights into the transcriptome of inflorescence divergence in *Panicum hallii* [30]. Moreover, previous study has profiled differentially expressed genes (DEGs) at three developmental stages of flower growth and compared them with those from vegetative seedling tissue using RNA-sequencing analysis, which confirmed that key genetic regulators, plant hormones, and cell-cycle genes were involved in barley inflorescence development [31]. In this study, we conducted transcriptomic, proteomic, and targeted phytohormone metabolomic analyses at three stages of inflorescence development, contrasting the two extreme materials to elucidate the regulatory mechanisms of inflorescence elongation in alfalfa.

## 2. Results

### 2.1. Distinct Phenotypes of Long and Short Inflorescences between Two Extreme Materials in Alfalfa

**Figure 1:**
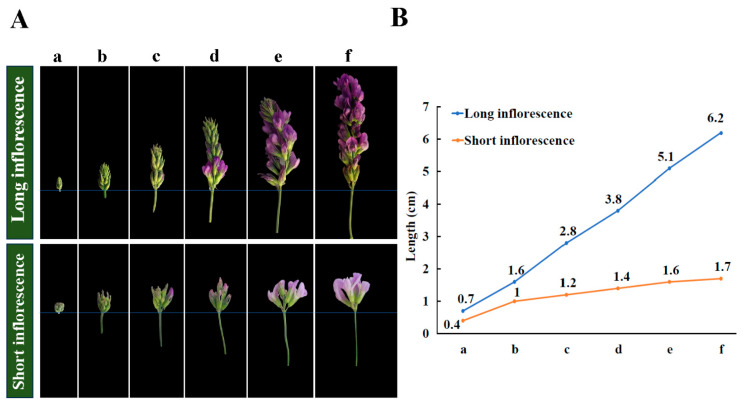
(**A**) Phenotypes of long and short inflorescence at the six developmental stages (a–f). (**B**) Values of length of long and short inflorescences at the six developmental stages (from the first floret at the base to the top).

In the present study, inflorescence development between long and short inflorescences was investigated in alfalfa. We classified the developmental process of the inflorescence into six stages, namely, inflorescence growth, including early budding stage ‘a’, full budding stage ‘b’, early flowering stage ‘c’, flowering stage 1 ‘d’, flowering stage 2 ‘e’, and full flowering stage ‘f’ (Figure 1A). As shown in Figure 1B, long inflorescence continuously elongated throughout the growth period; the length extended from 0.7 cm of ‘a’ to 6.2 cm of ‘f’. It displayed that the length difference was not prominent in the short inflorescence; the length extended from 0.4 cm of ‘a’ to 1.6 cm of ‘f’ (Figure 1B). The results revealed that long inflorescences had a stronger ability for peduncle elongation than short inflorescences in alfalfa, which provided adequate space for more florets to grow and perform photosynthesis. Notably, the inflorescence underwent pollination and fruiting when it reached full flowering, and the inflorescence peduncle almost stopped elongating and reached its maximum length. Based on these results, we speculated that the lack of ability for elongation during inflorescence development and most of the small flower buds blooming in a short amount of time were the major reasons for the structural formation of the short inflorescence. Conversely, the length of the long inflorescence continuously increased during inflorescence development; the florets gradually bloomed from the base, and the inflorescence peduncle elongated sustainably. The growth strategy of short inflorescences even led to the death of flower buds and florets owing to insufficient space, time, and nutrition for development, whereas the growth strategy of long inflorescences was able to support the growth and development of all florets and produce more seeds.

### 2.2. Genetic and Proteomic Analysis of Two Extreme Materials at the Three Developmental Stages

**Figure 2:**
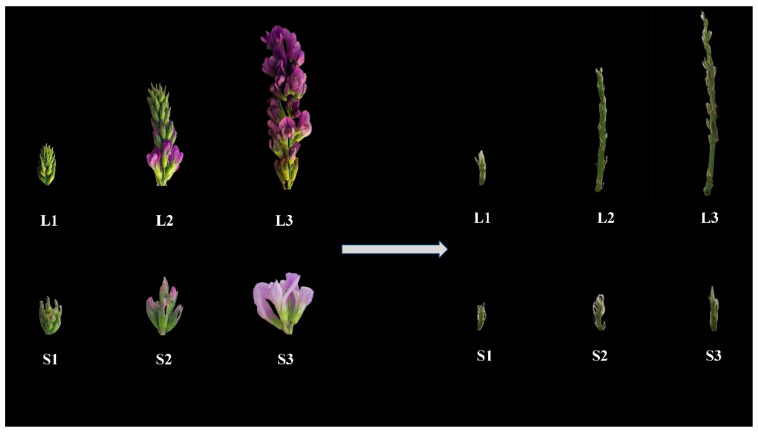
Phenotypes of long and short inflorescence peduncles at the three developmental stages, named L1, L2, L3, S1, S2, and S3, respectively.

**Figure 3:**
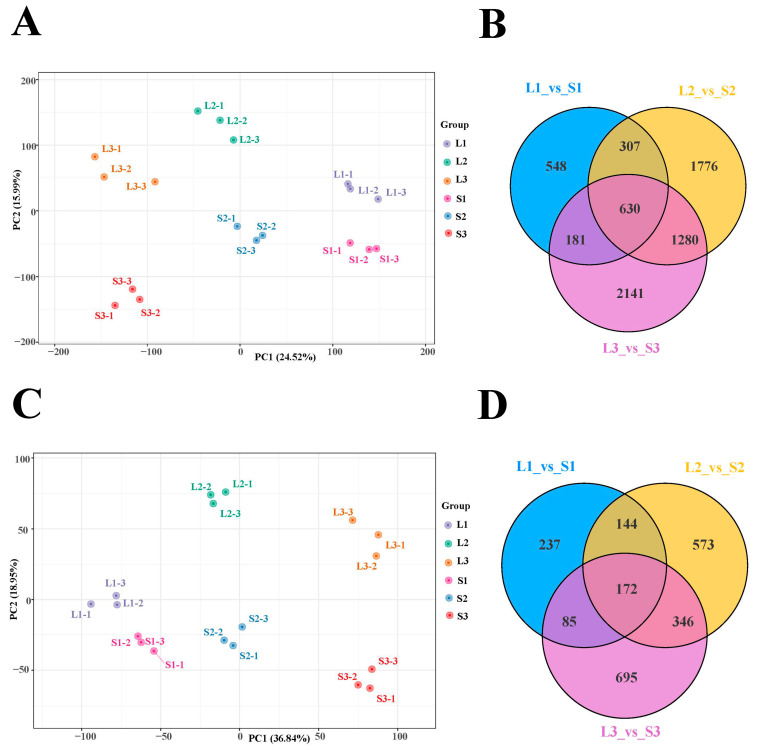
(**A**) PCA plot analysis of transcriptome; the x-axis represents principal component 1 (PC1); the y-axis represents principal component 2 (PC2). (**B**) Venn diagram of DEGs among three groups. (**C**) PCA plot analysis of proteome; the x-axis represents principal component 1 (PC1); the y-axis represents principal component two (PC2). (**D**) Venn diagram of DEPs among three groups. L1-1, L1-2, and L1-3 represent the three replicates of L1, as do the other samples.

To precisely elucidate the potential molecular mechanism of inflorescence elongation in alfalfa, we conducted a comparative transcriptome in two extreme materials at three developmental stages: the full budding stage of long and short inflorescences (L1 and S1), early flowering stage of long and short inflorescences (L2 and S2), and full flowering stage of long and short inflorescences (L3 and S3); the inflorescences were removed from the florets and the remaining inflorescence peduncles were used as experimental material (Figure 2). We detected a total of 127.56 Gb of clean data after removing low-quality reads, with more than 6 Gb of clean reads per library. The percentage of Q30 bases was, on average, 92%. No less than 73% of the clean reads from 18 samples could be mapped to the reference genome; this was indicative of the high quality of the transcriptome dataset (Table S2). Principal component analysis (PCA) showed an obvious separation among the six samples, and the contribution rates of PC1 and PC2 were 24.52% and 15.99%, respectively (Figure 3A), suggesting that significant differences between the six samples and the experiment could be reliable. For simplicity, we hereafter refer to the comparison of L1 vs. S1 as L1_S1, L2 vs. S2 as L2_S2, L3 vs. S3 as L3_S3, L2 vs. L1 as L2_L1, L3 vs. L2 as L3_L2, L3 vs. L1 as L3_L1, S2 vs. S1 as S2_S1, S3 vs. S2 as S3_S2, and S3 vs. S1 as S3_S1. Among groups, 1666 DEGs in L1_S1, 3993 in L2_S2, and 4232 in L3_S3 were identified at an FDR of < 0.05 and |log_2_Fold Change| ≥ 1 (Figure 3B). Additionally, we selected 11 key genes to perform qRT-PCR to determine the accuracy and reliability of the transcriptome datasets. The relative expression levels of these genes were similar to the RNA-Seq results, indicating that the RNA-Seq data could be trusted in this study (Figure S1).

Proteomic analysis was used to identify protein differences between the two extreme materials at three developmental stages. Similarly, PCA revealed that the proteomes were significantly separated among the six samples (Figure 3C), indicating that the proteome datasets could be trusted. We obtained and quantified 11,570 proteins from six samples. Among these proteins, we detected 638, 1235, and 1298 DEPs in L1_S1, L2_S2, and L3_S3, respectively (Figure 3D).

### 2.3. Enrichment Analysis of Integrative Genes and Proteins in Two Extreme Materials at the Three Developmental Stages

**Figure 4:**
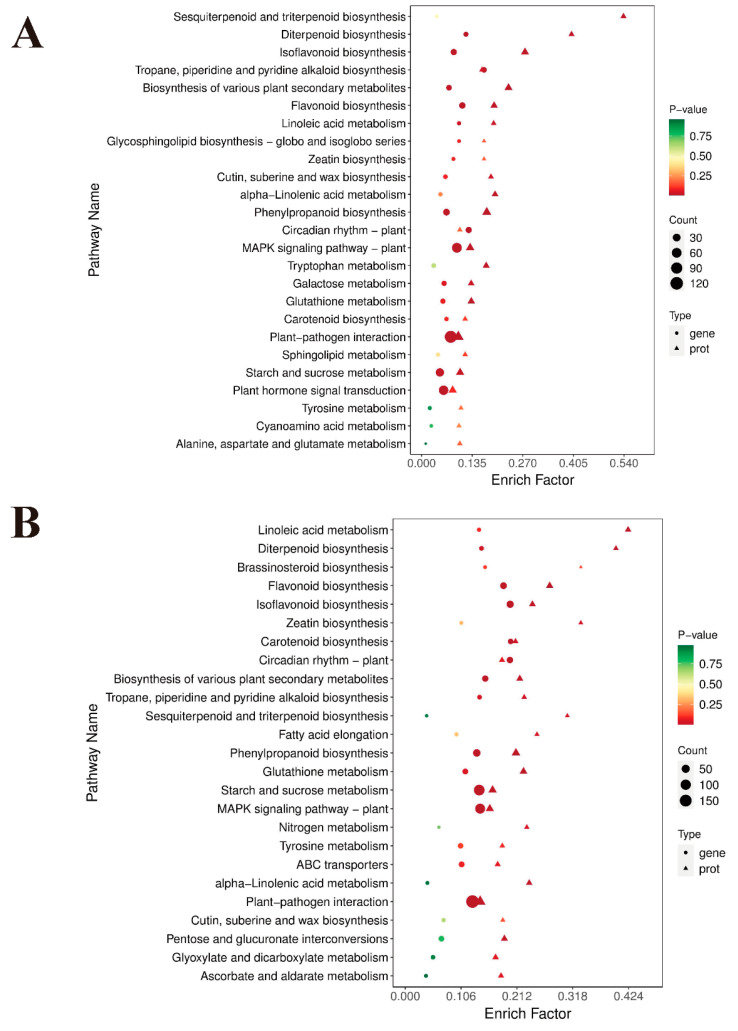

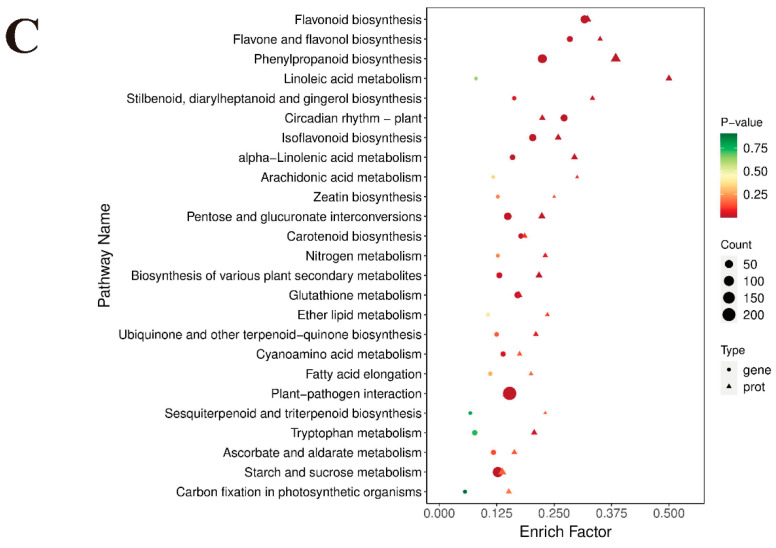
KEGG enrichment analysis of (**A**) L1_S1, (**B**) L2_S2, and (**C**) L3_S3 based on the integrative profiles of transcriptome and proteome. Color changes (red to green) represent low to high *p*-values. The area size of the circle and triangle represents the number of genes and proteins enriched in a pathway, respectively; the larger the area, the more there are.

We conducted comprehensive transcriptome and proteome analyses to elucidate the underlying mechanisms of inflorescence elongation. GO term analysis indicated that genes and proteins commonly participated in three predominant categories: biological processes; cellular components; and molecular functions. Among them, some essential GO terms were discovered to be correlated with inflorescence growth and development, including phenylpropanoid biosynthetic process, lignin biosynthetic process, plant-type cell wall, lignin catabolic process, plant-type secondary cell wall biogenesis, phloem development, flavonoid biosynthetic process, and other terms (Figure S2). Additionally, KEGG enrichment revealed that a large number of differentially regulated genes and proteins were enriched in the top 20 pathways associated with plant organ elongation and development in L1_S1, L2_S2, and L3_S3, such as phenylpropanoid biosynthesis, flavonoid biosynthesis, starch and sucrose metabolism, nitrogen metabolism, plant hormone signal transduction, and hormone biosynthesis-related pathways (Figure 4A–C), which might be closely related to inflorescence elongation.

### 2.4. Integrated Analysis of Genes and Proteins Associated with Lignin Biosynthesis

**Figure 5:**
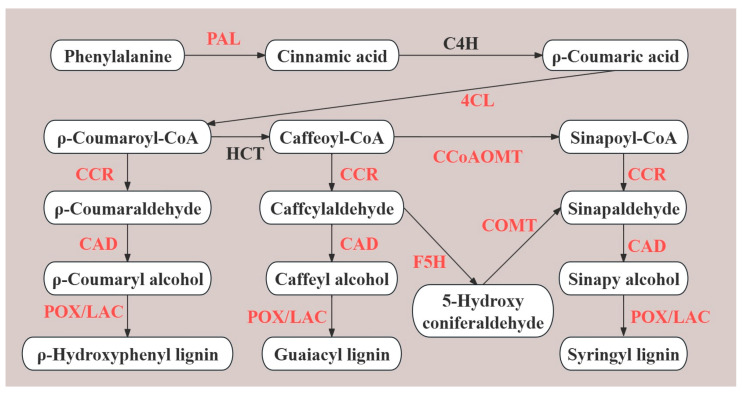
Structural genes and proteins participated in the lignin biosynthesis pathway between long and short inflorescence; red words represented that key enzyme participated in the lignin biosynthesis pathway in L1_S1, L2_S2, or L3_S3. *PAL*, L-phenylalanine ammonia-lyase; *C4H*, cinnamate 4-hydroxylase; *4CL*, 4-coumarate: CoA ligase; *HCT*, hydroxycinnamoyl CoA: shikimate hydroxycinnamoyl transferase; *CCoAOMT*, caffeoyl-CoA O-methyltransferase; *CCR*, cinnamoyl-CoA reductase; *CAD*, cinnamyl alcohol dehydrogenase; *F5H*, ferulate 5-hydroxylase; *COMT*, caffeic acid/5-hydroxyconiferaldehyde 3-O-methyltransferase; and *POX*/*LAC*, peroxidase/laccase.

The phenylpropanoid biosynthesis pathway produces lignin as an end product in vascular plants and is closely correlated with the growth of plant stems, roots, inflorescence peduncles, and other prop tissues. From the integrative profiles of the transcriptome and proteome, we found that phenylpropanoid biosynthesis was a crucial pathway related to the structural formation of the inflorescence. Furthermore, 24 DEGs and 24 DEPs, 49 DEGs and 29 DEPs, and 81 DEGs and 53 DEPs were detected in L1_S1, L2_S2, and L3_S3, respectively. To screen key genes and proteins related to the structural formation of long inflorescences, we established a lignin biosynthesis pathway (Figure 5). Furthermore, we detected that many genes and proteins were commonly upregulated in the lignin biosynthesis of key enzymes in the three comparison groups, including *POX*/*LAC* in L1_S1; *4CL*, *COMT*, *CAD,* and *POX*/*LAC* in L2_S2; and *PAL*, *4CL*, *CCoAOMT*, *CCR*, *CAD*, *F5H*, *COMT,* and *POX*/*LAC* in L3_S3 (Table S3), which might be crucial genes and proteins involved in lignin accumulation in long inflorescences.

### 2.5. Targeted Phytohormone Metabolome of Two Extreme Materials at the Three Developmental Stages

**Figure 6:**
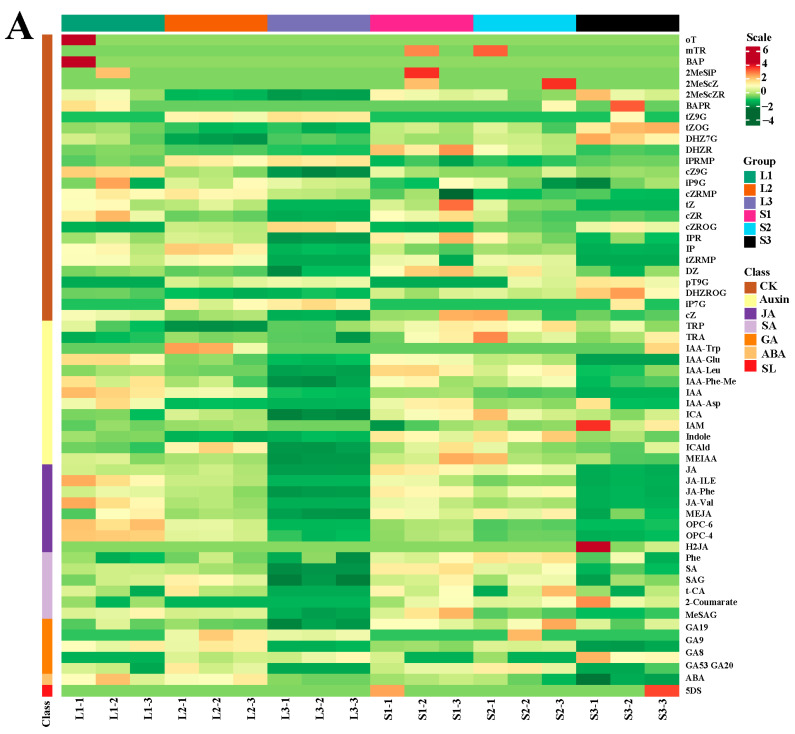

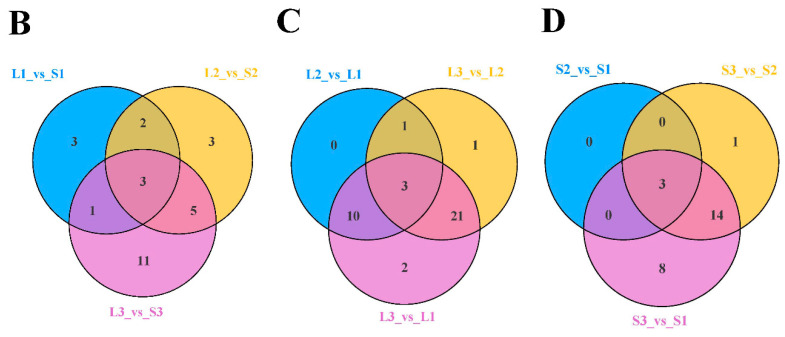
(**A**) Compounds class heatmap of phytohormone DAMs; L1-1, L1-2, and L1-3 represent the three replicates of L1, as do the other samples; color changes (green to red) represent low to high accumulation. Venn maps of (**B**) L1_S1, L2_S2, and L3_S3; (**C**) L2_L1, L3_L2, and L3_L1; (**D**) S2_S1, S3_S2, and S3_S1 based on targeted phytohormone analysis.

Plant hormones play critical roles in the development of tissues and organs [25], including auxins (IAA), cytokinin (CK), abscisic acid (ABA), jasmonate (Ja), salicylic acid (SA), gibberellin (GA), ethylene (ETH), strigolactone (SL), and melatonin (MLT). Based on the integrative results of transcriptome and proteome analyses, we found that plant hormone signal transduction and hormone biosynthesis-related pathways were closely associated with inflorescence elongation. To clarify the accumulation of plant hormones in all samples, we performed a targeted phytohormone metabolome analysis of the two extreme materials at three developmental stages. The heatmap is shown in Figure 6A, and 9, 13, 20, 14, 26, 36, 3, 18, and 25 DAMs were identified in L1_S1, L2_S2, L3_S3, L2_L1, L3_L2, L3_L1, S2_S1, S3_S2, and S3_S1, respectively (Figure 6B–D).

Compared with three developmental stages of long and short inflorescence, we identified one (indole-3-acetic acid, Log_2_ fold-change value = 1.18) and two (indole-3-acetic acid, Log_2_ fold-change value = Inf; indole-3-acetyl-L-tryptophan, Log_2_ fold-change value = 2.44) auxins that were significantly up-accumulated in L1_S1 and L2_S2, respectively. Quantitative analysis showed that a high content of indole-3-acetic acid (IAA) was maintained in both L1 (473.66 ng/mL) and L2 (328.06 ng/mL). These results suggest that IAA may play an essential role in inflorescence elongation. Moreover, we found that two GAs (GA9, Log_2_ fold-change value = 1.11; GA53, Log_2_ fold-change value = 2.10) and one GA (GA9, Log_2_ fold-change value = Inf) were prominently up-accumulated in L2_S2 and L3_S3, respectively. Based on the morphological characteristics of inflorescences and the function of GAs, we speculated that GA9 and GA53 might be closely related to inflorescence elongation, expansion of the peduncle diameter, and floret blooming. Additionally, some up-accumulated CKs were detected in L1_S1 (BAPR, Log_2_ fold-change value = Inf; cZRMP, Log_2_ fold-change value = 2.01) and L2_S2 (tZ9G, Log_2_ fold-change value = Inf; iPRMP, Log_2_ fold-change value = 1.54; iP7G, Log_2_ fold-change value = Inf); these CKs might play an essential role in inflorescence growth at the full budding and early flowering stages. In addition, differential accumulations of Jas and SA were detected in L1_S1, L2_S2, and L3_S3, which may be involved in the growth and development of inflorescences.

Notably, most phytohormones were significantly down-accumulated in L3_L2, L3_L1, S3_S2, and S3_S1 (Table S4). The results suggested that the metabolic activity of the inflorescence peduncle at the full flowering stage was significantly weaker than that at the full budding and early flowering stages, which was consistent with the developmental regularity in which the inflorescence peduncle stopped elongating and mainly played a role in structural support and nutrient transport.

### 2.6. Integrated Analysis of Genes, Proteins, and Metabolites Related to Plant Hormone Biosynthesis

**Figure 7:**
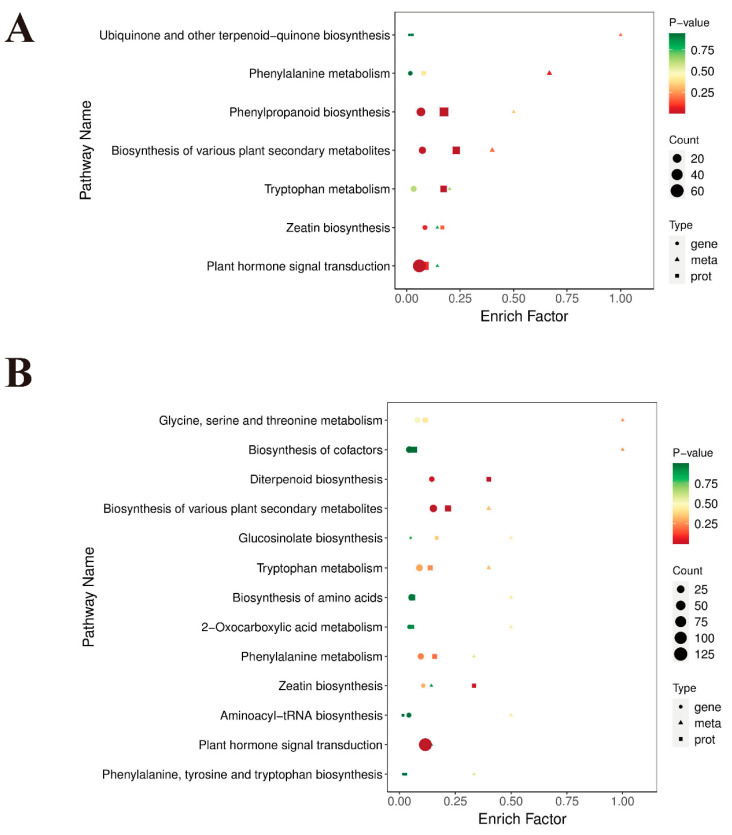
KEGG enrichment analysis of (**A**) L1_S1 and (**B**) L2_S2 based on integrative profiles of transcriptome, proteome, and metabolome. Color changes (red to green) represent low to high *p*-values. The area size of the circle and triangle represents the number of genes and proteins enriched in a pathway, respectively; the larger the area, the more there are.

Based on the results above, we conclude that IAA, GA, and CK are closely correlated with inflorescence elongation at the full budding and early flowering stages. To further investigate the underlying mechanism of plant hormone biosynthesis in L1_S1 and L2_S2, we conducted an integrative analysis of the transcriptome, proteome, and metabolome of long and short inflorescences. KEGG enrichment analysis showed that some differentially regulated genes, expressed proteins, and accumulated metabolites were enriched in pathways related to plant hormone biosynthesis in L1_S1 and L2_S2, including tryptophan metabolism (ko00380), diterpenoid biosynthesis (ko00904), and zeatin biosynthesis (ko00908) (Figure 7A,B). 

**Figure 8:**
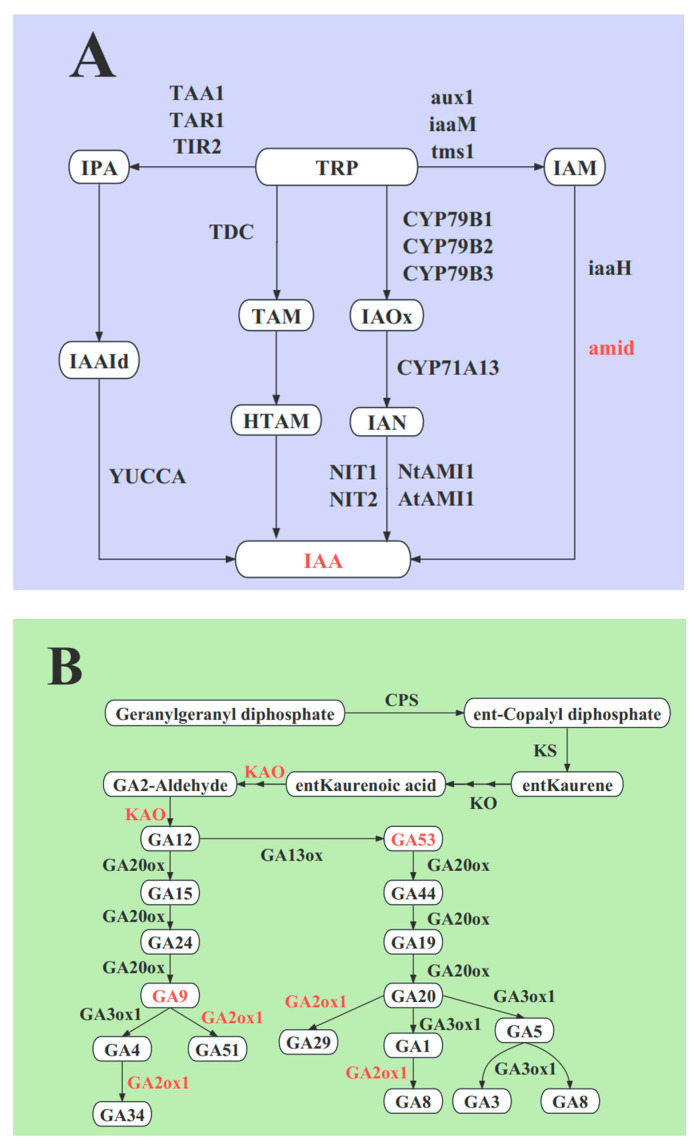
Key enzymes and metabolites participated in (**A**) auxin and (**B**) GA biosynthesis pathways; red words represented crucial enzymes or metabolites. The red font in the box represents the high accumulation of metabolites, and the red font next to the arrow represents the commonly high expression of genes and proteins in the long inflorescences compared to the short inflorescences.

In tryptophan metabolism (ko00380), the integrative analysis showed that one amiE (amidase) protein (*A0A072TU58*, Log_2_ fold-change value = 1.32) and two *amiE* genes (*novel.12346*, Log_2_ fold-change value = 2.65) were commonly upregulated in L1_S1, and we identified that the content of indole-3-acetic acid was higher in L1 than in S1 (Figure 8A), suggesting that the gene *novel.12346* might be transcribed and translated into protein *A0A072TU58*, and eventually, indole-3-acetic acid was synthesized. 

In diterpenoid biosynthesis (ko00904), integrative analysis showed that one *KAO* (ent-kaurenoic acid monooxygenase) protein (*A0A072V5K8*, Log_2_ fold-change value = 0.72) and two *KAO* genes (*MsG0580024673.01*, Log_2_ fold-change value = 1.22; *MsG0580024666.01*, Log_2_ fold-change value = 1.61) were commonly upregulated in L2 compared to S2 (Figure 8B), suggesting that they participated in the synthesis of GA53 and GA9. In addition, two *GA2ox* (gibberellin 2beta-dioxygenase) proteins (*G7IJL7*, Log_2_ fold-change value = 0.65; *G7K254*, Log_2_ fold-change value = 1.13) and one *GA2ox* gene (*MsG0580029209.01*, Log_2_ fold-change value = 1.01) were upregulated in L2_S2 (Figure 8B), which might be closely correlated with the accumulation of other GAs in L2.

In addition, we detected some differentially expressed genes and proteins related to IAA, GA, and CK biosynthesis in the transcriptome and proteome, respectively, such as *YUCCA*, *TAA1*, *GA20ox*, *IPT*, and other enzymes that might also participate in regulating inflorescence growth.

### 2.7. Integrated Analysis of Genes, Proteins, and Metabolites Related to Plant Hormone Signal Transduction

**Figure 9:**
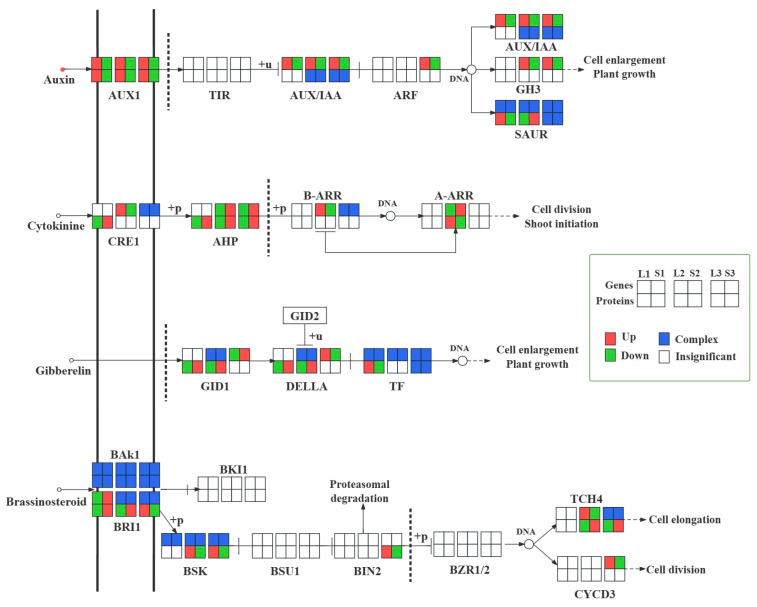
IAA, CK, GA, and BR signal transduction diagram. The six rows of small squares represent the three comparison groups, including L1_S1, L2_S2, and L3_S3. The genes and proteins are located on upper and lower sides, respectively. Red, green, blue, and white boxes represent up-regulation, down-regulation, complex, and insignificant, respectively.

Moreover, we identified many DEGs and DEPs involved in plant hormone signal transduction between long and short inflorescences, which were mainly enriched in IAA, CK, GA, and BR signaling pathways. As shown in Figure 9, we examined the IAA, CK, GA, and BR signaling pathways. In plant hormone signal transduction among three comparisons, indole-3-acetic acid was a unique differential metabolite detected in L1_S1 and L2_S2. Previous studies have revealed that the auxin signaling pathway was closely related to plant growth and organ elongations [19]. In general, we discovered that many DEGs involved in plant hormone signal transduction were enriched in the auxin signaling pathway, and most DEGs involved in the auxin signaling pathway were upregulated in long inflorescences rather than in short inflorescences, according to transcriptome datasets. Therefore, we identified that auxin signaling played a more essential role than other signal transduction pathways in regulating the elongation of inflorescences. By further investigating the pathways of auxin signaling pathway based on the transcriptome, proteome, and metabolome, we identified a total of 18, 23, and 17 upregulated DEGs, 2, 3, and 3 upregulated DEP, and 1, 1, and 0 upregulated DAMs in L1_S1, L2_S2, and L3_S3, respectively. Commonly upregulated genes and proteins were identified in the three comparison groups: *AUX1*; *AUX*/*IAA*; and *SAUR* (Figure 9). These results suggest that IAA induces the expression of genes involved in the auxin signaling pathway and further induces the expression of downstream genes associated with inflorescence peduncle elongation.

In addition, we found that some DEGs and DEPs were enriched in the CK, GA, and BR signaling pathways between long and short inflorescences (Figure 9). For example, encoding *DELLA* genes and proteins were commonly downregulated in L2_S2 (Figure 9), which might also affect inflorescence elongation.

### 2.8. Weighted Gene and Protein Co-Expression Network Analysis

**Figure 10:**
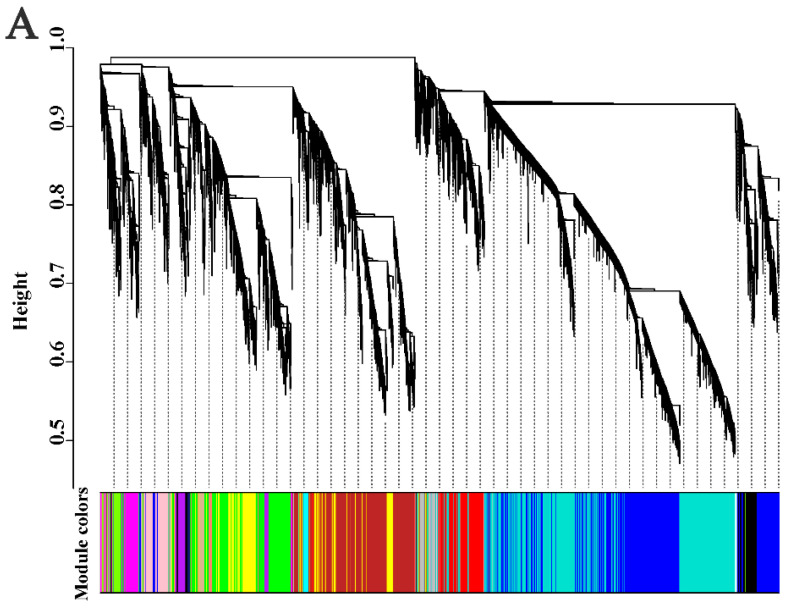

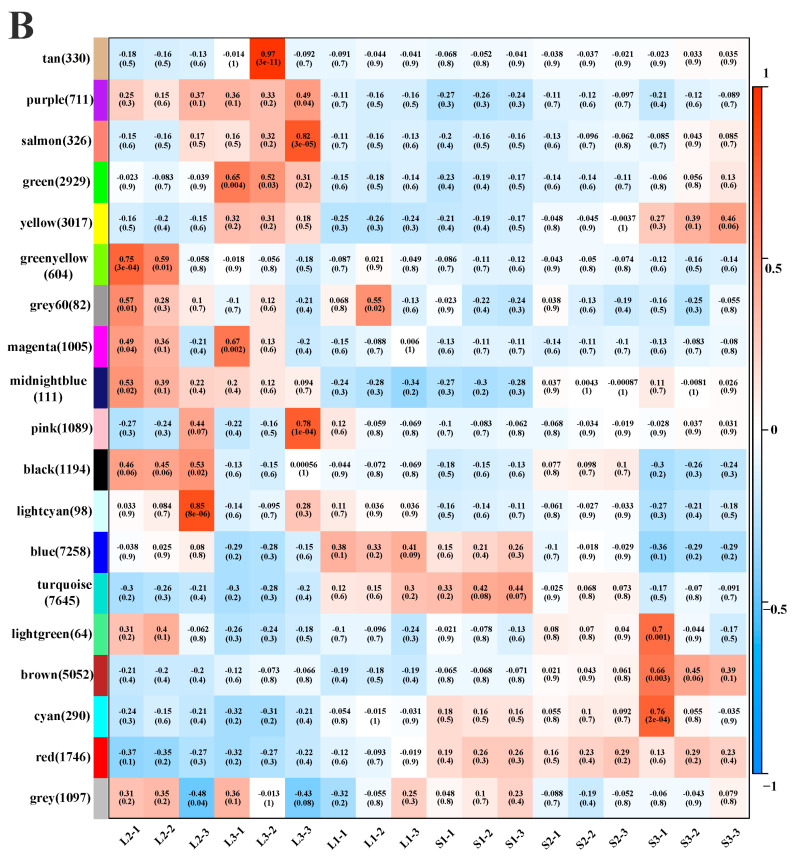

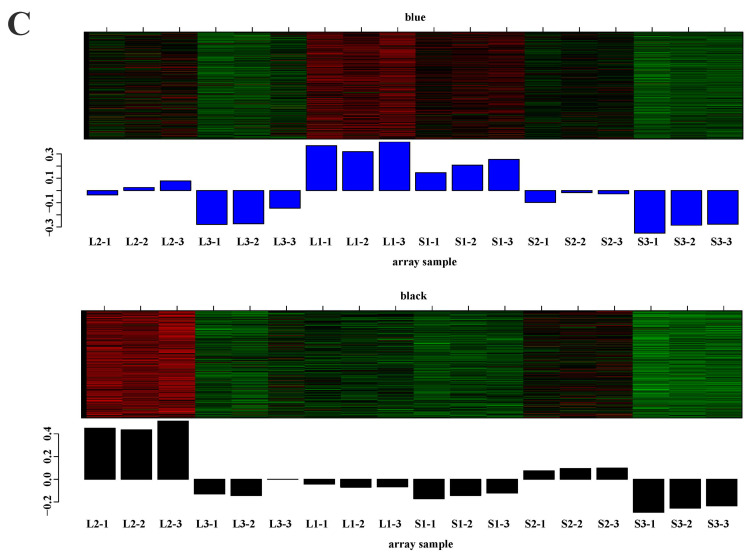
(**A**) Dendrogram displaying module eigengenes detected by WGCNA and clustering dendrogram of expressed genes. (**B**) The heatmap of correlation coefficient between samples and modules with positive and negative correlations is shown in red and blue, respectively. (**C**) Eigengene expression analysis of blue and black module.

To further search for candidate genes highly correlated with inflorescence elongation, we performed weighted gene co-expression network analysis (WGCNA). As a result, a dynamic hierarchical tree cut showed that 19 modules with similar gene expression patterns were detected by WGCNA, which are defined using different colors (Figure 10A). Furthermore, we screened several modules that were highly correlated with each sample from the WGCNA results (R > 0.3) (Figure 10B). Concerning L1, a blue module was selected. For L2, a black module was selected. For L3, purple and green modules were selected. For S1, a turquoise module was selected. For S2, no module was selected. For S3, a brown module was selected. 

Based on morphological studies of the alfalfa raceme, we confirmed that the gene expression of the inflorescence peduncle at the full budding and early flowering stages played a more critical role in affecting the growth and development of the inflorescence peduncle than that at the full flowering stage. Eigengene expression analysis of the blue and black modules is shown in Figure 10C, indicating that patterns were closely correlated with the full budding and early flowering stages, respectively. Therefore, genes in the blue and black modules can be speculated to be associated with inflorescence elongation. Among the genes belonging to the blue and black groups, we identified 28 genes that were related to lignin biosynthesis, IAA and GA biosynthesis, and IAA signaling pathway (Table S5), which might be candidate genes involved in inflorescence development.

Similarly, WPCNA (weighted protein co-expression network analysis) results indicated that 11 modules had similar protein expression patterns, and yellow and purple modules (1308 and 76 proteins, respectively) were recognized as crucial modules associated with the elongation of the inflorescence peduncle (Figure S3). From these two modules, we screened a total of six proteins involved in lignin and GA biosynthesis, including *POX*/*LAC* (*G7IC23*, *G7JJ71*, and *G7LDV0*), *CAD* (G7JFC2), *KAO* (G7IJL7), and *GA2ox* (*G7K254*) (Table S6) that might be key proteins associated with inflorescence peduncle elongation.

### 2.9. Transcription Factors Analysis

**Figure 11:**
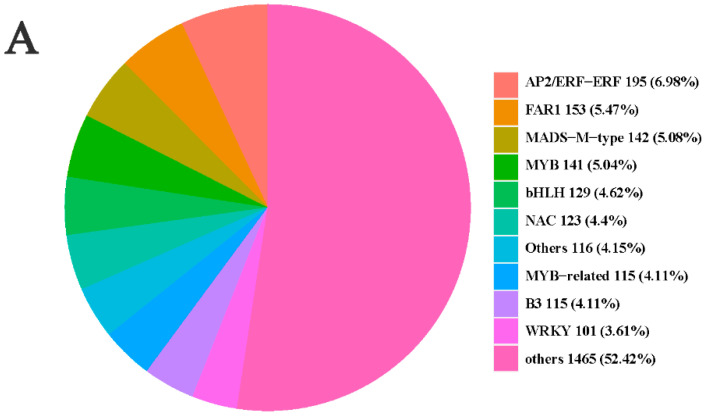

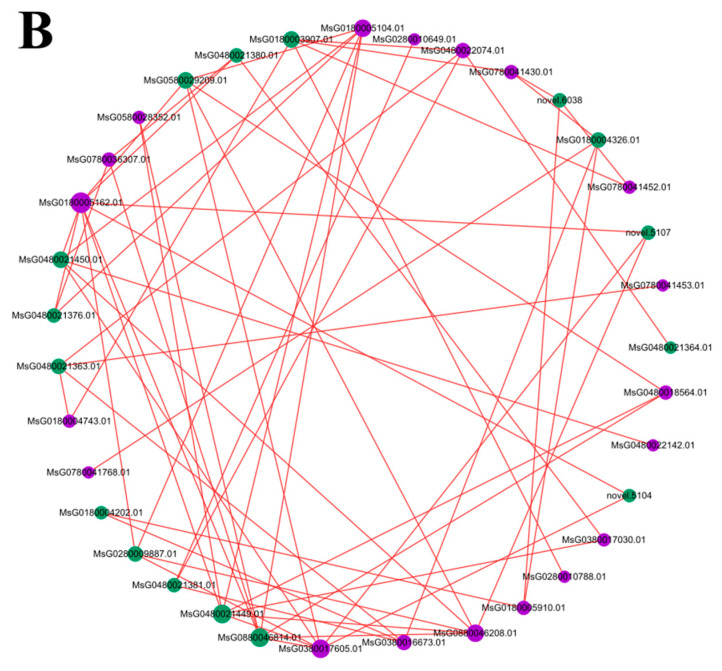
(**A**) TF family classification among genes. (**B**) Correlation analysis between candidate genes and TFs; purple and green circles represent TFs and candidate genes, respectively.

Transcription factors (TFs) play essential roles in regulating the expression of structural and regulatory genes. In this study, a total of 2796 TFs were identified among genes and classified into 92 TFs families, including *AP2*/*ERF*-*ERF* (195), *FAR1* (153), *MADS-M-*type (142), *MYB* (141), *bHLH* (129), *NAC* (123), Others (116), *MYB*-related (115), B3 (115), and *WRKY* (101) (Figure 11A). Among the TFs families, some TFs might play a crucial role in the growth and elongation of the inflorescence peduncle. Subsequently, we screened the differentially expressed TF genes in the blue and black modules to identify the critical TFs that regulated candidate genes. In L1_S1 and L2_S2, 24 differentially expressed TFs were identified in two modules (FPKM > 2). Furthermore, we performed a correlation analysis between these TFs and 28 candidate genes (|r| > 0.7, *p* < 0.05). Nineteen TFs were positively correlated with 16 structural genes ((Figure 11B and Table 1). In the lignin biosynthesis pathway, we identified 13 TFs that were positively related to five structural genes, including *MYB* (four), *bHLH* (two), and *NAC* (one). In the plant hormone biosynthesis and signal transduction, we identified 17 TFs that were positively associated with 11 candidate genes, including *MYB* (seven), *bHLH* (four), *NAC* (two), *GRAS* (one), and *HB* (one). These TFs may play an important role in regulating structural and regulatory genes and eventually prompt the elongation of the inflorescence peduncle.

## 3. Discussion

The architecture of plant inflorescence determines flower and seed growth and development, and length is an essential character influencing the morphological structure of alfalfa inflorescences, which is also closely correlated with seed production of alfalfa [8]. To clarify the potential mechanism of alfalfa inflorescence elongation, phenylpropanoid biosynthesis was selected as a critical pathway between the two extreme materials by analyzing the integrative transcriptomic and proteomic results. Combined with the targeted phytohormone metabolome, we identified key genes, proteins, and metabolites associated with plant hormone biosynthesis and signal transduction based on omics analysis.

The phenylpropanoid pathway is important for lignin synthesis and is closely associated with the elongation of plant organs [32]. In this study, we found that many differentially expressed genes and proteins were enriched in the phenylpropanoid pathway between long and short inflorescences, which suggested that lignin biosynthesis played an essential role in inflorescence structure formation. *PAL*, *C4H*, and *4CL* are upstream enzymes in the phenylpropanoid biosynthesis pathway, and their expression levels in plants significantly affect lignin accumulation [33,34]. *HCT*, *F5H*, *COMT*, and *CCoAOMT* affect the synthesis of lignin monomers, and the inhibition of these enzymes significantly reduces the accumulation of lignin [35,36]. Downstream lignin biosynthesis determines the synthesis of different lignins, which are associated with four enzymes, including *CCR*, *CAD*, and *POD*/*LAC* [37,38,39]. In the present study, we screened many commonly upregulated genes and proteins related to enzymes involved in lignin biosynthesis in L1_S1, L2_S2, and L3_S3, including *PAL*, *4CL*, *CCR*, *CAD*, *CCoAOMT*, *F5H*, *COMT*, and *POD*/*LAC*. High expression levels of these genes or proteins may contribute to the synthesis of related enzymes, thereby increasing lignin accumulation in long inflorescences.

Phant hormones are pivotal for the growth of the inflorescence peduncle. The auxin indole-3-acetic acid (IAA) is a crucial promoter of plant tissue growth. Previous studies have shown that IAA played a crucial role in promoting stem and root elongation. Consistent with our findings, we found that the IAA content of the full budding and flowering stages was significantly upregulated in long inflorescences compared to short inflorescences, suggesting that IAA might be a key phytohormone contributing to the elongation of alfalfa inflorescences. *Pollmann* discovered that amidase plays a role in catalyzing the conversion of IAM to IAA in *Arabidopsis thaliana* [40]. In this study, we identified that one gene and one protein, amid, were commonly upregulated in L1_S1, suggesting that amidases might play an important role in IAA accumulation in long inflorescences. Subsequently, IAA acts on related receptors and prompts the expression of auxin response genes, eventually resulting in its biological effects. *AUX1* plays a critical role in auxin transport. A previous report demonstrated that *AtAUX1* mutations led to *tAUX1* mutations, attenuating auxin trafficking in *Arabidopsis* seedlings and altering IAA distribution in young leaf and root tissues [41]. *Aux*/*IAA*, an important functional gene, participates in plant growth and development by regulating the downstream genes of the auxin signaling pathway [42]. *SAUR* gene family can respond early in auxin induction; overexpression of *AtSAUR63* elongated the hypocotyls and stamen filament of the transgenic plants in *Arabidopsis* [43]. In this study, we discovered that most DEGs and DEPs maintained higher expression levels in long inflorescences than in short inflorescences. In addition, we further identified that many genes and proteins encoding *AUX1*, *Aux*/*IAA*, and *SAUR* were commonly up- or down-regulated in L1_S1, L2_S2, and L3_S3, revealing that *AUX1*, *Aux*/*IAA,* and *SAUR* might play an essential role in regulating the elongation of long inflorescences.

Gibberellins facilitate cell wall extension, stem development, and tissue growth [44]. The targeted phytohormone metabolome showed that the concentrations of GA9 and GA53 were significantly higher in L2 than in S2, suggesting that GA9 and GA53 might be involved in the elongation and growth of inflorescences at the early flowering stage. *KAO* is an upstream enzyme of the GA biosynthesis pathway. In the present study, we identified that two *KAO* genes and one *KAO* protein maintained higher expression in L2 than in S2, which might be involved in the accumulation of GA and contribute to the growth and elongation of inflorescences at the early flowering stage. Most studies have demonstrated that *DELLA* proteins inhibited plant growth by binding to transcription factors related to the regulation of plant growth [45,46,47]. In this study, we discovered that *DELLA* proteins were downregulated in L1_S1 and L2_S2, and *DELLA* genes maintained low expression levels in L2 compared to S2. The results revealed that *DELLA* proteins might play a negative role in the elongation and development of alfalfa inflorescences. 

Additionally, some pathways were connected to plant growth and development based on an integrated analysis of the transcriptome and proteome, such as starch and sucrose metabolism, nitrogen metabolism, and flavonoid biosynthesis [48,49,50,51]. These results show that these pathways might also be involved in the structural formation of inflorescences. Previous studies revealed that *MYB*, *NAC*, and *bHLH* TFs participated in the regulation of lignin biosynthesis [52,53]. In this study, we identified that four *MYB*, two *bHLH*, and one *NAC* TFs were positively correlated with candidate genes associated with lignin biosynthesis based on a correlation analysis, revealing that these TFs might play an essential role in regulating structural genes related to lignin accumulation.

## 4. Materials and Methods

### 4.1. Plant Materials

Alfalfa (*Medicago sativa*) samples with long and short inflorescences were cultivated at the Grassland Institute of the Chinese Academy of Agricultural Sciences in Hohhot (40°58′ N, 111°78′ E). All samples were collected when the alfalfa reached the blooming stage, as we could obtain all samples during this period. We selected three alfalfas with long inflorescence and nine alfalfas with short inflorescence as candidate materials; inflorescences were collected based on the developmental stages and inflorescence lengths (1.5–2.0 cm for the full budding stage of long inflorescence ‘L1’, 3.5–4.0 cm for the early flowering stage of long inflorescence ‘L2’, 6.0–6.5 cm for the full flowering stage of long inflorescence ‘L3’, 0.8 to 1.2 cm for the full budding stage of short inflorescence ‘S1’, 1.2–1.5 cm for the early flowering stage of short inflorescence ‘S2’, and 1.5–2 cm for the full flowering stage of short inflorescence ‘S3’). We removed all flowers and retained the inflorescence peduncle from the first floret, from the base to the top. Three biological replicates were obtained for the six samples, and the inflorescence peduncle for each replicate was heavier than 3.0 g.

### 4.2. Transcriptome Sequencing and Data Analysis

The total RNA of the inflorescence peduncle (L1, L2, L3, S1, S2, and S3) was extracted by ethanol precipitation and CTAB-PBIOZOL. RNA purity and integrity were analyzed using an Agilent 2100 Bioanalyzer and a Qubit 2.0 Fluorometer. Eighteen cDNA libraries were sequenced using an Illumina Sequencing 6000 platform. After the low-quality sequences were removed, clean reads were assembled using fastp software (fastp v0.19.4). All non-redundant transcripts were mapped using the *Medicago sativa* reference genome (https://figshare.com/articles/dataset/Medicago_sativa_genome_and_annotation_files/12623960 (accessed on 10 August 2023)). DESeq2 software (DESeq2 v3.19) was used to determine the differential expression profiles among the samples. Subsequently, we obtained notably differential genes with a false discovery rate (FDR < 0.01) and fold change (FC ≥ 2). Enrichment analysis was conducted based on a hypergeometric test, with pathway-based hypergeometric distribution checking for Kyoto Encyclopedia of Genes and Genomes (KEGG) and Gene Ontology (GO) term-based profiles. Finally, a weighted gene co-expression network analysis (WGCNA) was performed using the varFilter function in the R genefilter package. The correlation network diagram was conducted by using R version 3.5.1.

### 4.3. Proteomic Analysis

Proteins were extracted from the samples using acetone precipitation. Protein samples extracted from the inflorescence peduncle were incubated in L3 buffer (0.15 M, pH 8.0) containing 1% SDS, 100 mMTris-HCl, 7 M urea, 2 M thiourea, 1 mM PMSF, and 2 mM EDTA and ultrasonically cracked on ice for 10 min. After the protein solution was obtained by centrifuging the supernatant, we added 4× volume of frozen acetone into the protein solution, precipitated at −20 °C overnight, and centrifuged at 4 °C to obtain the precipitate. After obtaining the precipitate, we washed it with cold acetone, dissolved it in 8 M urea, and measured protein concentration. Equal amounts of protein from each sample were subjected to tryptic digestion. Then, we used tryptic to digest equal content of proteins from each sample, added 8 M urea to 200 μL to the supernatants, reduced with 10 mM DTT for 45 min at 37 °C, and alkylated with 50 mM iodoacetamide (IAM) for 15 min in a dark room at 20 °C. The protein precipitate was air-dried and resuspended in 200 μL of 25 mM ammonium bicarbonate solution and 3 μL of trypsin (Promega) and digested overnight at 37 °C after adding 4× volume of chilled acetone, precipitated at −20 °C for 2 h and centrifuged. Subsequently, we desalted for peptides, dried and concentrated, using a C18 cartridge, vacuum concentration meter, and vacuum centrifugation, respectively, and eventually re-dissolved in 0.1% (*v*/*v*) formic acid.

### 4.4. Hormone Analysis

The LC-MS/MS analysis was conducted using a Q Exactive HF-X mass spectrometer combined with an Easy-nLC 1000 system (Thermo Fisher Scientific, Waltham, Massachusetts, USA). LC–MS/MS was performed as previously described by Zhu et al. (2022) [5]. The protein sequences obtained above were blasted in the specified tax ID of the nr database using the BLASTP algorithm, with the principle that proteins with the same or similar amino acid sequences share similar functions. Annotations from the mapped protein hits, mainly GO terms (including Biological Process, Cellular Component, and Molecular Function) and KEGG pathway information, were transferred to the original submitted proteins.

Quantification of endogenous auxins, cytokinins (CKs), abscisic acid (ABA), jasmonates (Jas), salicylic acid (SA), gibberellin (Gas), ethylene (ETH), strigolactones (SLs), and melatonin (MLT) was performed by Wuhan Metware Biotechnology Co., Ltd. (Wuhan, China) using an LC–MS/MS. The samples (15 mg) were dissolved in 1 mL of methanol/water/formic acid (15:4:1, *v*/*v*/*v*) and frozen in liquid nitrogen. Ten microlitres of the internal standard mixed solution (100 ng/mL) was added to the extract as internal standard (IS) for quantification. Subsequently, the supernatant was transferred to clean plastic microtubes after the liquid was vortexed (10 min), centrifugated (12,000 r/min, 5 min, and 4 °C), followed by evaporation to dryness and dissolved in 100 μL 80% methanol (*v*/*v*) and filtered for further LC-MS/MS analysis. The UPLC and ESI-MS/MS conditions were described by Niu et al. [54]. The detected metabolites were annotated using the KEGG compound database (http://www.kegg.jp/kegg/compound/ (accessed on 12 August 2023)).

### 4.5. qRT-PCR Analysis

The RNA-Seq results were confirmed by using quantitative real-time PCR. The primers were designed using Primer3 (https://primer3.ut.ee/ (accessed on 8 January 2024)). Primers used are listed in Supplementary Table S1. The actin gene (MsG0380015289.01) was selected as the reference gene in this study because of its high and steady expression levels in all samples based on transcriptome data. All results were obtained from three repetitions.

## 5. Conclusions

In this study, the regulatory mechanisms of inflorescence elongation in alfalfa were investigated using transcriptome, proteome, and targeted phytohormone metabolome analyses. Specifically, we analyzed the developmental processes of long and short inflorescences in alfalfa and used the three developmental stages of two inflorescences as experimental materials. Compared to short inflorescences, we found that IAA, GA, and CK played crucial roles in regulating peduncle elongation according to the metabolome results. Additionally, based on omics analyses, we detected candidate genes and proteins correlated with lignin biosynthesis, GA biosynthesis, auxin biosynthesis, and signaling pathways. Moreover, TFs related to lignin biosynthesis, GA biosynthesis, auxin biosynthesis, and signaling pathways were identified using correlation analysis. The results of this study highlight the developmental processes and the potential mechanisms underlying inflorescence elongation in alfalfa and provide a theoretical foundation for germplasm innovation.

## Figures and Tables

**Table 1:** The identified transcription factors in the blue and black modules.

Gene ID	Module	NR	TF-Family
MsG0180005104.01	blue	transcription factor bHLH30	bHLH
MsG0180005162.01	blue	transcription factor bHLH30	bHLH
MsG0280011275.01	blue	dehydration-responsive element-binding protein 3	AP2/ERF-ERF
MsG0380015775.01	blue	LOB domain-containing protein 25	LOB
MsG0380017605.01	blue	transcription factor MYB3R-1	MYB
MsG0480018564.01	blue	receptor protein kinase-like protein ZAR1	S1Fa-like
MsG0480022142.01	blue	homeobox-leucine zipper protein HOX3	HB-other
MsG0580025575.01	blue	transcription factor bHLH93 isoform X1	bHLH
MsG0580028352.01	blue	zinc finger protein CONSTANS-LIKE 9	C2C2-CO-like
MsG0780036307.01	blue	transcription factor MYB16	MYB
MsG0780038789.01	blue	probable WRKY transcription factor 49	WRKY
MsG0880042918.01	blue	transcription factor MYC1	bHLH
MsG0880046208.01	blue	scarecrow-like protein 28	GRAS
MsG0180004743.01	black	transcription factor MYB61 isoform X1	MYB
MsG0180005910.01	black	zinc finger CCCH domain-containing protein 15	C3H
MsG0280010649.01	black	transcription factor MYB14	MYB-related
MsG0280010788.01	black	NAC domain-containing protein 73	NAC
MsG0380016673.01	black	hypothetical protein DVH24_022336	NAC
MsG0380017030.01	black	transcription factor bHLH94	bHLH
MsG0480022074.01	black	myb transcription factor	MYB
MsG0780041430.01	black	transcription factor MYB61	MYB
MsG0780041452.01	black	transcription factor MYB61	MYB
MsG0780041453.01	black	transcription factor PIF3 isoform X1	bHLH
MsG0780041768.01	black	ethylene-responsive transcription factor ERF023	AP2/ERF-ERF

## Data Availability

All data are open and available. The raw data are available in the NCBI database (BioProject ID PRJNA1014246) (https://www.ncbi.nlm.nih.gov/sra/ (accessed on 8 September 2023)).

## Supplementary Materials

The following supporting information can be downloaded at https://www.mdpi.com/article/10.3390/ijms25126497/s1.
