# Supplementary material for: Integrated Multi-Omics Analysis to Reveal the Molecular Mechanisms of Inflorescence Elongation in Medicago sativa
Source: Int J Mol Sci. 2024 Jun 12;25(12):6497. doi: 10.3390/ijms25126497 (PMC11203646; doi:10.3390/ijms25126497)
Supplement: Supplementary file 1 [file ijms-25-06497-s001.zip › ijms-3047717-supplementary/Supplymental Figures--IJMS.pdf]

Supplemental Figures

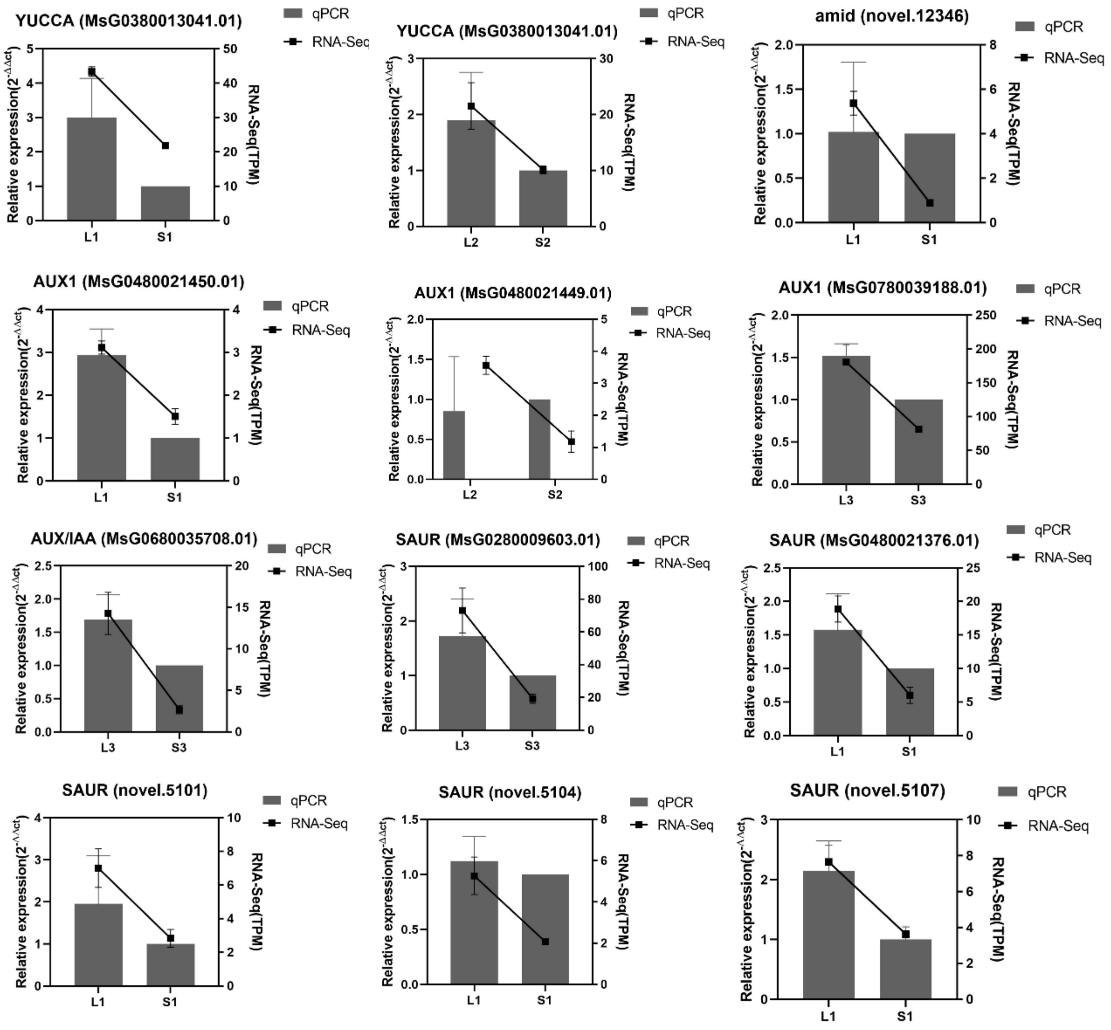

Supplemental Figure S1: qRT-PCR analysis of candidate gene expression patterns in L1_S1, L2_S2 and L3_S3.

A

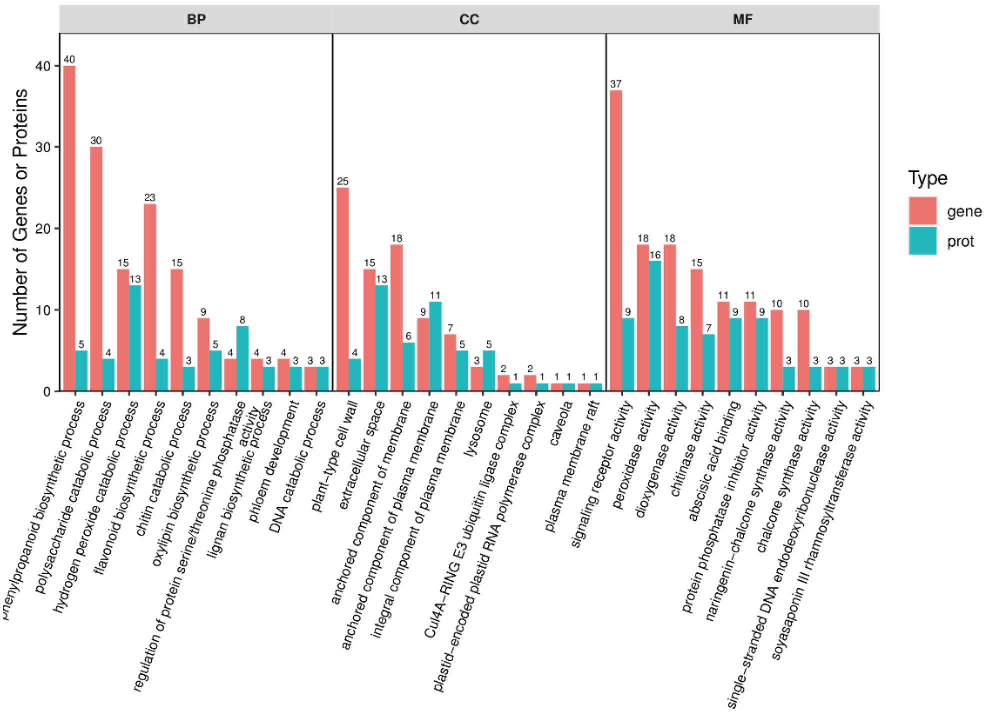
BP
CC
MF
Number of Genes or Proteins
Type
gene
prot
phenylpropanoid biosynthetic process
polysaccharide catabolic process
hydrogen peroxide catabolic process
flavonoid biosynthetic process
chitin catabolic process
oxylipin biosynthetic process
regulation of protein serine/threonine phosphatase activity
lignan biosynthetic process
phloem development
DNA catabolic process
plant–type cell wall
extracellular space
anchored component of membrane
anchored component of plasma membrane
integral component of plasma membrane
lysosome
Cul4A–RING E3 ubiquitin ligase complex
plastid–encoded plastid RNA polymerase complex
caveola
plasma membrane raft
signaling receptor activity
peroxidase activity
dioxygenase activity
chitinase activity
abscisic acid binding
protein phosphatase inhibitor activity
naringenin–chalcone synthase activity
chalcone synthase activity
single–stranded DNA endodeoxyribonuclease activity
soyasaponin III rhamnosyltransferase activity

B

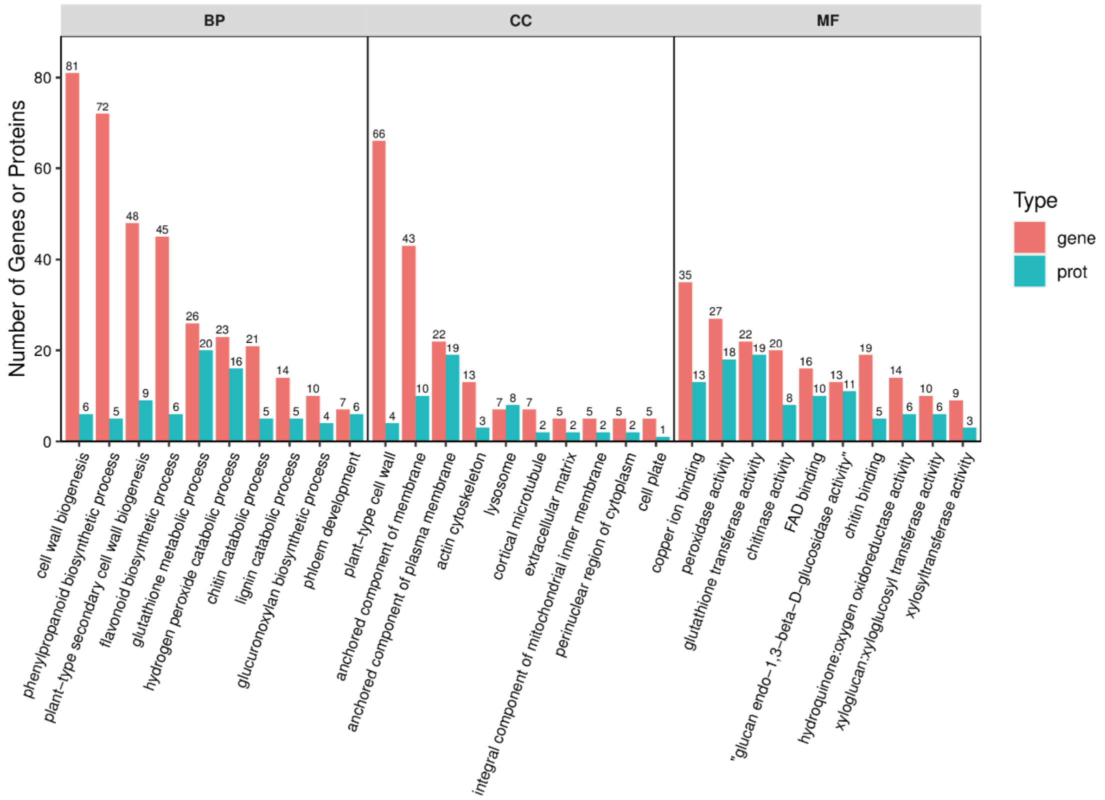
BP
CC
MF
Number of Genes or Proteins
Type
gene
prot
cell wall biogenesis
phenylpropanoid biosynthetic process
plant–type secondary cell wall biogenesis
flavonoid biosynthetic process
glutathione metabolic process
hydrogen peroxide catabolic process
chitin catabolic process
lignin catabolic process
glucuronoxylan biosynthetic process
phloem development
plant–type cell wall
anchored component of membrane
anchored component of plasma membrane
actin cytoskeleton
lysosome
cortical microtubule
extracellular matrix
integral component of mitochondrial inner membrane
perinuclear region of cytoplasm
cell plate
copper ion binding
peroxidase activity
glutathione transferase activity
chitinase activity
FAD binding
"glucan endo–1,3–beta–D–glucosidase activity"
chitin binding
hydroquinone:oxygen oxidoreductase activity
xyloglucan:xyloglucosyl transferase activity
xylosyltransferase activity

C

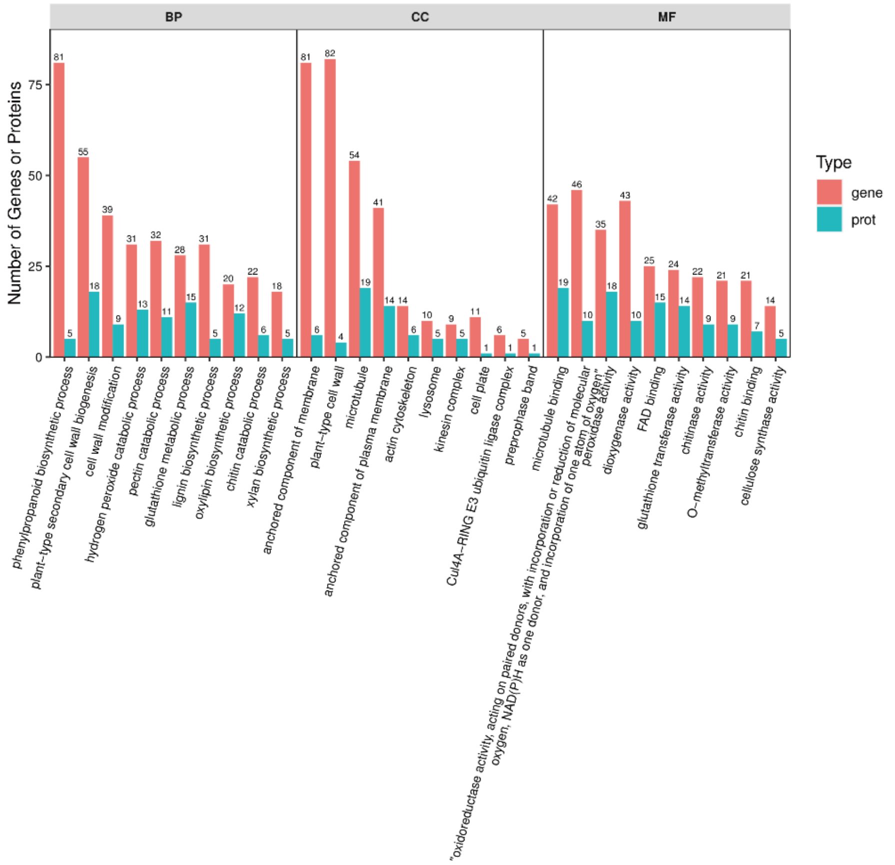

Supplemental Figure S2: GO enrichment map in (A) L1_S1, (B) L2_S2, and (C) L3_S3.

A

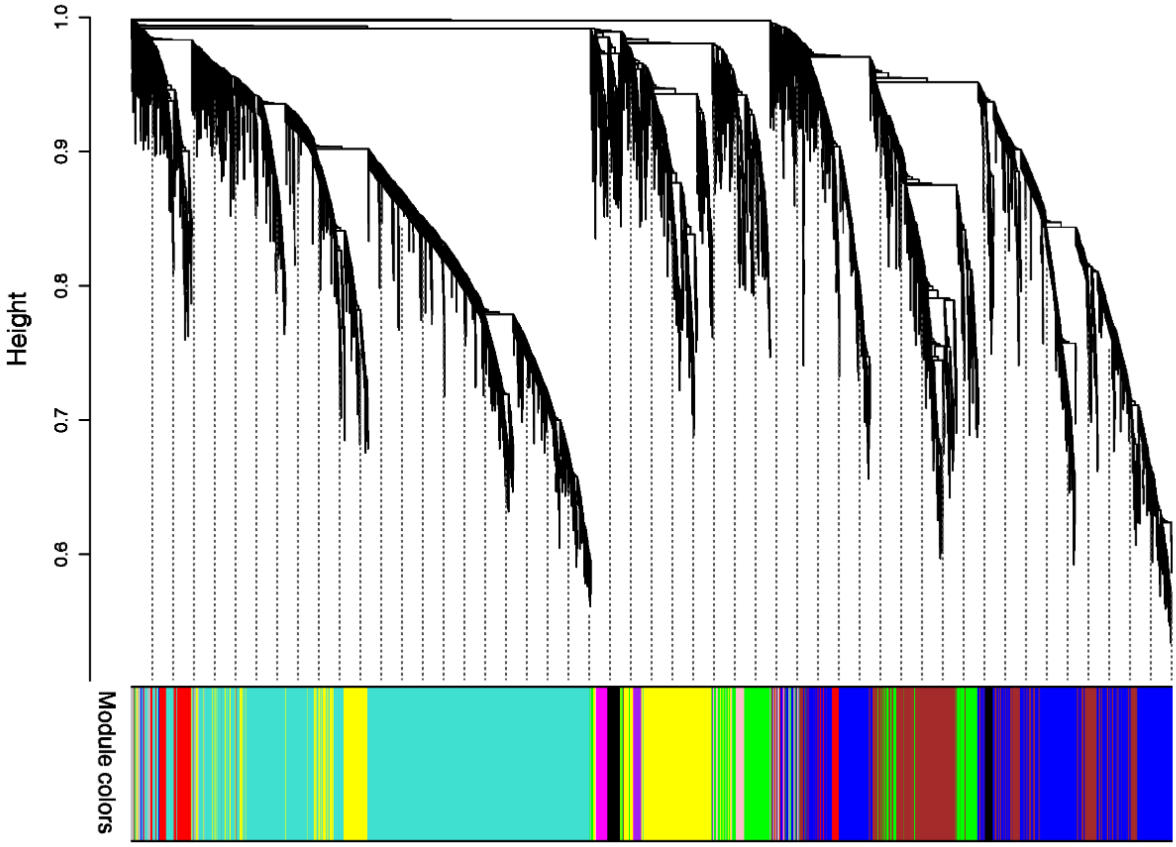

B

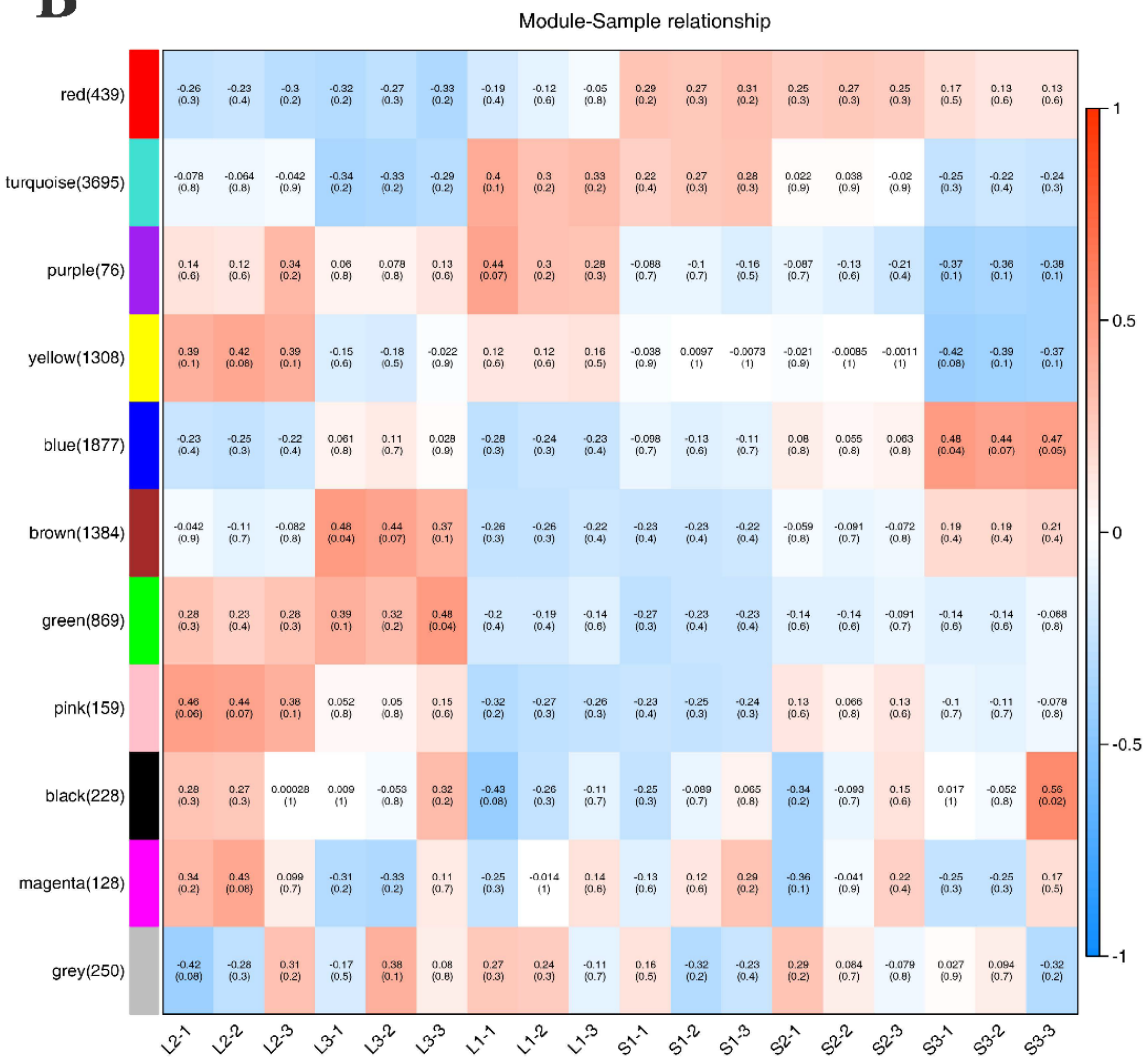

Supplemental Figure S3: (A) Dendrogram displaying modules detected by WGPNA and clustering dendrogram of expressed proteins. (B) The heatmap of correlation coefficient between samples and modules with a positive and negative correlations showed in red and blue, respectively.
